# Response to “Hidradenitis suppurativa electronic medical record (EPIC) tool: A convenient tool of hidradenitis suppurativa specialty clinics”

**DOI:** 10.1016/j.jdin.2025.01.003

**Published:** 2025-01-21

**Authors:** Terri Shih, Katrina Lee, Vivian Y. Shi, Jennifer L. Hsiao

**Affiliations:** a Department of Dermatology, Kaiser Permanente Los Angeles Medical Center, Los Angeles, California; b Department of Dermatology, University of Southern California, Los Angeles, California; c Department of Dermatology, University of Arkansas for Medical Sciences, Little Rock, Arkansas

**Keywords:** hidradenitis suppurativa, multidisciplinary, specialty clinic

We appreciate Wendland et al’s recognition of our article,^1^ which introduced a paper-based intake form for hidradenitis suppurativa (HS) specialty clinics. We concur that integrating an HS-specific provider assessment within the electronic medical record (EMR) can optimize clinic flow and commend their creation of an “HS Assessments” tool made available to all EPIC users. We would like to further the discussion by sharing our perspectives on the potential utility of EMR-based tools.

EMR tools offer numerous advantages, including minimizing risk for error (ie illegible handwriting or transcription errors) and data loss (ie incorrectly scanned, misplaced, or shredded forms) and reducing paper waste. Wendland et al’s HS nurse assessment contains valuable data, and we propose that patient-directed questions could be distributed online during electronic check-in to save time in a busy clinic. We also wonder about the time required to complete the HS provider assessment tool. Its comprehensiveness is reminiscent of clinical trial tools and may be fitting for research purposes in HS specialty clinics. Due to time constraints, the tool may be challenging to implement regularly during a general dermatology clinic.

A more simplified HS provider assessment tool for everyday use in busy dermatology clinics would be very useful. The tool can be designed to utilize physical exam findings in the note to facilitate tracking of treatment responses and auto-generate text phrases in provider notes to aid with insurance prior authorization requirements. A simplified assessment tool would likely avoid the need to count individual lesions of different anatomic areas.

With the emergence of artificial intelligence in EMR,^2^ there are even more opportunities for EMRs to not only optimize the visit intake, but also the visit close-out. Existing artificial intelligence programs can already record the patient encounter and summarize patient education into after-visit summaries. Having a flare management plan is a key component of HS care,^3^ and an EMR tool could be designed to pre-build a flare action plan for providers to edit and provide to patients. EMR tools also have the potential to automate screening for HS comorbidities and coordinate care, utilizing surveys to auto-generate referrals to appropriate specialties (ie PHQ-2 or PHQ-9 for depression and subsequent referral to behavioral health), and prompting clinic notes to be sent to a patient’s primary care provider when applicable.

While HS specialty clinics (www.hs-foundation.org/hs-specialty-clinics)^4^ are a great resource for patients with severe, recalcitrant disease, our goal is for all dermatologists to view themselves as HS experts. Wendland et al’s HS Assessments tool represent an excellent starting point for HS specialty clinics, and a more streamlined provider assessment tool would be welcome to optimize the HS patient visit in a general dermatology clinic. We hope that further development of HS-specific standardized EMR tools and expansion beyond EPIC will help clinicians save time, enhance patient care, and increase access to care by lowering barriers to effective clinic visits. Continued efforts to leverage EMR and technology are needed to optimize care, especially for our patients with complex management needs, such as those with HS.

## Conflicts of interest

Dr Shi is on the board of directors for the Hidradenitis Suppurativa Foundation, an advisor for the National Eczema Association, is a stock shareholder of Learn Health, and has served as an advisory board member, investigator, speaker, and/or received research funding from Sanofi Genzyme, Regeneron, AbbVie, Genentech, Eli Lilly, Novartis, SUN Pharma, LEO Pharma, Pfizer, Incyte, Dermavant, Boehringer Ingelheim, Almirall, Alumis, Aristea Therapeutics, Menlo Therapeutics, Dermira, Burt’s Bees, Galderma, Kiniksa, UCB, Ceraclere, Bain Capital, Target-PharmaSolutions, Castle Bioscience, Altus Lab/cQuell, MYOR, Polyfins Technology, GpSkin and Skin Actives Scientific. Dr Hsiao is on the board of directors for the Hidradenitis Suppurativa Foundation, and has served as an advisor, investigator, and/or speaker for AbbVie, Aclaris, Amgen, Boehringer Ingelheim, Galderma, Incyte, Novartis, Sanofi Regeneron, UCB. Drs Lee and Shih have no conflicts of interest to declare.
